# Application of FGD-BCEL loss function in segmenting temporal lobes on localized CT images for radiotherapy

**DOI:** 10.3389/fonc.2023.1204044

**Published:** 2023-10-05

**Authors:** Xiaobo Wen, Bing Liang, Biao Zhao, Xiaokun Hu, Meifang Yuan, Wenchao Hu, Ting Liu, Yi Yang, Dongming Xing

**Affiliations:** ^1^ The Affiliated Hospital of Qingdao University, Qingdao University, Qingdao, China; ^2^ Department of Radiotherapy, Yunnan Cancer Hospital, the Third Affiliated Hospital of Kunming Medical University, Kunming, Yunnan, China; ^3^ School of Pharmacy, Qingdao University, Qingdao, China; ^4^ Qingdao Cancer Institute, Qingdao University, Qingdao, China; ^5^ School of Basic Medicine, Qingdao University, Qingdao, China; ^6^ Affiliated Hospital of Qingdao University, Interventional Medicine Center, Qingdao, Shandong, China; ^7^ Department of Endocrinology Qilu Hospital (Qingdao), Cheeloo College of Medicine, Shandong University, Qingdao, Shandong, China; ^8^ School of Life Sciences, Tsinghua University, Beijing, China

**Keywords:** U-Net model, deep learning, medical-image segmentation, temporal lobe, radiotherapy, loss function

## Abstract

**Objectives:**

The aim of this study was to find a new loss function to automatically segment temporal lobes on localized CT images for radiotherapy with more accuracy and a solution to dealing with the classification of class-imbalanced samples in temporal lobe segmentation.

**Methods:**

Localized CT images for radiotherapy of 70 patients with nasopharyngeal carcinoma were selected. Radiation oncologists sketched mask maps. The dataset was randomly divided into the training set (*n* = 49), the validation set (*n* = 7), and the test set (*n* = 14). The training set was expanded by rotation, flipping, zooming, and shearing, and the models were evaluated using Dice similarity coefficient (DSC), Jaccard similarity coefficient (JSC), positive predictive value (PPV), sensitivity (SE), and Hausdorff distance (HD). This study presented an improved loss function, focal generalized Dice-binary cross-entropy loss (FGD-BCEL), and compared it with four other loss functions, Dice loss (DL), generalized Dice loss (GDL), Tversky loss (TL), and focal Tversky loss (FTL), using the U-Net model framework.

**Results:**

With the U-Net model based on FGD-BCEL, the DSC, JSC, PPV, SE, and HD were 0.87 ± 0.11, 0.78 ± 0.11, 0.90 ± 0.10, 0.87 ± 0.13, and 4.11 ± 0.75, respectively. Except for the SE, all the other evaluation metric values of the temporal lobes segmented by the FGD-BCEL-based U-Net model were improved compared to the DL, GDL, TL, and FTL loss function-based U-Net models. Moreover, the FGD-BCEL-based U-Net model was morphologically more similar to the mask maps. The over- and under-segmentation was lessened, and it effectively segmented the tiny structures in the upper and lower poles of the temporal lobe with a limited number of samples.

**Conclusions:**

For the segmentation of the temporal lobe on localized CT images for radiotherapy, the U-Net model based on the FGD-BCEL can meet the basic clinical requirements and effectively reduce the over- and under-segmentation compared with the U-Net models based on the other four loss functions. However, there still exists some over- and under-segmentation in the results, and further improvement is needed.

## 1 Introduction

Global cancer statistics show that the incidence and mortality rates of nasopharyngeal carcinoma (NPC) in Southeast and East Asia are at high levels, posing a severe threat to patients’ safety and life quality. Radiotherapy proved to be an important and effective treatment for NPC (1, 2). During radiation therapy for NPC, the temporal lobe is inevitably irradiated during irradiation due to its anatomical location and structure, thus causing different degrees of side effects (3). Temporal lobe injury (TLI) is the most common side effect of radiotherapy for NPC (4). It is one of the severe late complications affecting memory, neurocognitive function, physical function, emotion and language, and life quality (5). Some researchers have shown that the degree of temporal lobe damage in patients with NPC is affected by the maximum dose, with the incidence of TLI increasing by 2.6% for each 1 Gy increase when Dmax ≥ 64 Gy (6). Moreover, TLI correlates with the irradiated volume of the temporal lobe, and when temporal lobe necrosis (TLN) occurs, patients with V45 >15.1 cc are more likely to suffer from massive necrosis (7), causing positive results such as cognitive decline. Therefore, the temporal lobe must be precisely protected as organs at risk (OARs).

In current clinical treatment, radiation oncologists mainly delineate OARs manually, making the delineation subjective and experience-affected, and resulting in differences in the irradiated dose and volume of the temporal lobe, thus increasing the risk of TLN. With the rapid development of artificial intelligence, deep learning-based automatic delineation was gradually developed and applied in clinical work. Ibragimov et al. used convolutional neural networks (CNNs) for predictive segmentation of head and neck OARs, and they obtained segmentation results ranging from 37.4% DSC for the optic chiasm to 89.5% DSC for the mandible (8). Their results showed that most of the OARs could be accurately delineated, which showed that automatic delineation could avoid the influence of individual differences with more efficiency. Nevertheless, among the studies on OAR segmentation of NPC, some researches failed to include the temporal lobe as OAR (9), which makes the study on the automatic delineation of the temporal lobe on localized CT images inadequate. In addition, most current studies on automatic segmentation of the temporal lobe and related diseases were performed on MRI with few reports on big aperture localization CT for radiotherapy. Big-aperture CT images are characterized by big aperture, small row size, and relatively poor image pixels, which necessitates further studies on the automatic segmentation of the temporal lobe on CT images to observe the segmentation effect of deep learning.

The training effectiveness of a deep learning model hinges upon two critical factors: the model architecture and the choice of loss function (10). Adjusting the model architecture necessitates the redesign and training of the model, which can be a time-consuming and computationally intensive process (11). Furthermore, computers with low computational power cannot provide the required running environment for the training and application of the model with larger parameters (12–14). By contrast, modifying the loss function is a comparatively more practicable approach. The choice of loss function directly impacts the training process and convergence of the model (15). A well-suited loss function can guide the model to be optimized in the desired direction. Therefore, improvements based on the loss function have the potential for greater generalizability and applicability to a certain extent.

In recent years, CNNs have been widely used for medical image segmentation. Typically, these networks employ the cross-entropy loss function for training and model convergence. However, because of the same weight shared by all the samples, the cross-entropy loss function does not yield satisfactory results when dealing with the classification of class-imbalanced samples. For example, the temporal lobe occupies a relatively small proportion on localized CT images for radiotherapy due to its anatomical structural characteristics. With the traditional cross-entropy loss function, the model tends to predict the pixel points as background, leading to incorrect predictions of the temporal lobe. Consequently, the traditional cross-entropy loss function fails to perform well on the dataset. In addressing the issue of imbalanced samples in medical image segmentation, Milletari et al. (16) proposed a loss function called the Dice loss function in their study of V-net. This loss function is designed based on the Dice similarity coefficient (DSC) and allows direct comparison between predicted images and ground truth. However, its gradient provokes oscillation during the training, which affects the model’s accuracy. In addressing the challenges posed by unbalanced data, Sudre et al. (17) introduced the generalized Dice loss function (GDL) in their study. GDL aims to effectively balance the relationship between lesions and Dice coefficients. However, when dealing with highly unbalanced data, GDL may still exhibit some fluctuations. Additionally, while GDL addresses class imbalance by applying weights, incorrect weight selection and an excessive emphasis on certain classes at the expense of others can potentially impact the overall performance of the model. Similarly, Lin et al. (18) proposed a focal loss function to solve the problems caused by data class imbalance in the image domain. It improves the model’s accuracy in an imbalance sample by putting the difficulty of the sample classification in the first consideration and making the loss function focus on complex samples. However, the NAN value quickly occurs due to its oversized loss function. The Tversky loss function serves as an extension of the DL loss function, enabling effective adjustment of the balance between false positives and false negatives through the utilization of hyperparameters. On the other hand, the FTL loss function builds upon the concept of FL by incorporating power weighting to further refine this balance. However, both methods still rely on DL for refinement, resulting in potential issues with gradient stability and subsequent accuracy concerns. Moreover, the limited number of pixel points occupied by the upper and lower poles of the temporal lobe poses a challenge for the currently employed loss function. Consequently, it becomes necessary to develop a new loss function that can effectively address these challenges. In this study, we proposed a novel loss function called FGD-BCEL, in the light of weight assignments by FL, TL, and FTL loss functions. To assess its performance, we adopted the widely used U-Net model as the framework and employed five different loss functions (FGD-BCEL, DL, GDL, TL, and FTL) respectively to segment the temporal lobes on localized CT images for radiotherapy.

## 2 Methods

### 2.1 Dataset acquisition

The experimental dataset used in this study was obtained from 70 patients with NPC admitted to the radiotherapy department of Yunnan Cancer Hospital from May 2020 to September 2021. Each patient was simulated and positioned using Siemens large-aperture CT (Somatom Sensation Open, 24 rows, Φ85 cm) with a 5-mm or 3-mm layer thickness. Patient computed tomography scans contained the complete temporal lobe with an image resolution of 512 × 512. Mask maps were delineated by the radiation oncologists using the 3D slicer software, as shown in **Supplementary Figure 1**. In this study, the dataset was randomly divided into the training set (*n* = 49), validation set (*n* = 7), and test set (*n* = 14) with the proportion of 7:1:2. To improve the generalization and robustness of the model, we expanded the training set by rotation, flipping, zooming, and shearing. The expanded training set contained 2,094 temporal lobe CT slices.

### 2.2 Image preprocessing

Image preprocessing in this study is performed on the dataset, including HU value transformation, window width and window level adjustment, adaptive histogram equalization, and image normalization operations. The Simple ITK package automatically converts HU value transformation; window width and window level values are 160 and 80, respectively; and the image normalization operation normalizes the image range to [0,1].

### 2.3 Model architecture

**Figure 1:**
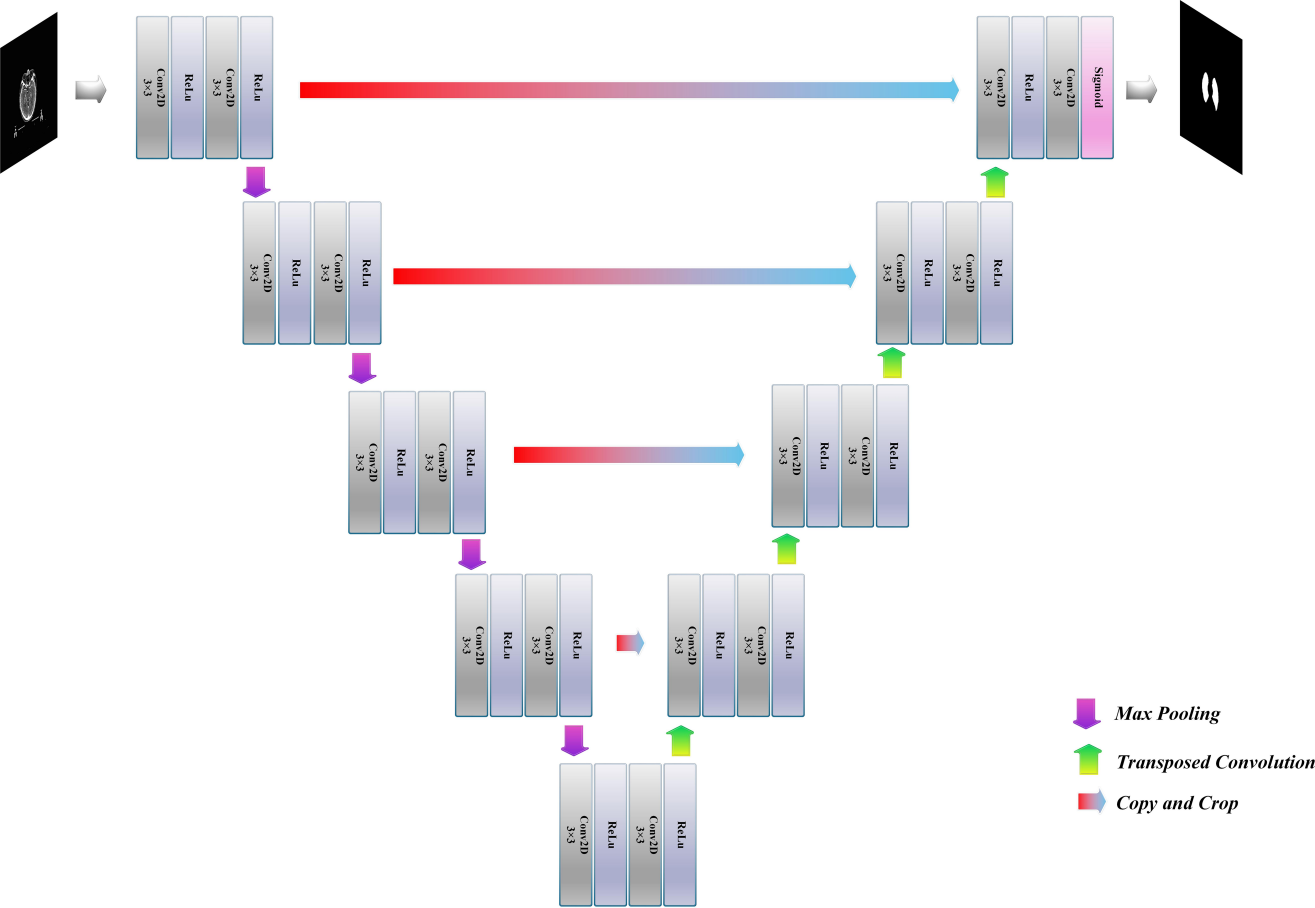
U-Net structure.

The U-Net architecture (19), the widely used model in the medical field, is used in this study. It consists of three parts: downsampling, upsampling, and “bridge” connections, as shown in **Figure 1**. The downsampling module on the left is mainly used for the initial extraction of temporal lobe features and the compression of images and features, also called Encode. The downsampling consists of four blocks of different-resolution images, each consisting of two 3 × 3 convolutional layers and a 2 × 2 max pooling layer. The convolutional layer extracts the temporal lobe features layer by layer, and the max pooling layer is used for image and feature compression. The arrow in the middle indicates the “bridge” connection. Its primary function is to copy and crop the feature map obtained by downsampling and upsampling, forming a feature map with deep and shallow information to achieve more effective segmentation. The right part is the upsampling module, mainly used for temporal lobe image size recovery and further feature extraction. The upsampling part also consists of four blocks of different-resolution images. Each block contains one 3 × 3 deconvolution layer and two 3 × 3 convolution layers. The deconvolution layer reduces the feature map size so that the final output size is the same as the original map.

In this study, the initial input of the model is a 512 × 512 × 1 temporal lobe CT image, and the output result is a 512 × 512 × 1 model prediction image. The ReLU activation function is used for upsampling and downsampling convolutional feature extraction, and the Sigmoid activation function is used for the final result output. In order to ensure that the corresponding features of downsampling and upsampling can be fused correctly, the image resolution size must be the same when the features are fused. Therefore, this paper adopts a complementary zero-fill approach in the convolution operation to ensure that the image remains constant in size during feature extraction at each resolution and removes the cropping step in the “bridge” connections.

### 2.4 Loss function

This paper combines GDL and focal loss and incorporates the binary cross-entropy (BCE) loss function to smooth the training process further. The paper proposes an FGD-BCEL with the formula provided in Equation (1). In this study, the GDL loss function replaced the traditional cross-entropy loss function and α is added to further regulate the weight of each pixel in the GDL loss function. The BCE loss function with weights is incorporated to regulate the smoothing of the loss function in the training process, to improve the model’s accuracy. To explore the effect of FGD-BCEL, this study employed the same U-Net framework with five loss functions, the proposed FGD-BCEL, DL (16), GDL (17), TL (20), and FTL (21), respectively, to segment the temporal lobe on localized CT images for radiotherapy.


(1) $$FGD-BCEL={\left(1-2\frac{\sum_{l}^{2}\omega_{l}\sum_{n}r_{\ln}\;p_{\ln}}{\sum_{l}^{2}\omega_{l}\sum_{n}r_{\ln}+p_{\ln}}\right)}^{\alpha }+\beta *BCE$$

The GDL loss function is in parentheses, where $r_{\ln}$ is the standard value of category l at the *n*th pixel, $p_{\ln}$ is the predicted pixel probability value, $\omega_{l}$ is the weight of each category, α is used to regulate the size of the weight of each pixel, and the value of this study is 0.75. BCE is the binary cross-entropy loss function, and *β* is used to regulate the weight of the BCE loss function and adjust the smoothing of the loss function during model training. The value is 0.7 in this study.

### 2.5 Model evaluation metrics

In this study, DSC, JSC, PPV, SE, and HD, which are more commonly used in medical image segmentation, were used to further evaluate the generalization ability and segmentation accuracy of the U-Net models based on the five different loss functions respectively. The DSC and JSC are calculated in Equations (2) and (3).


(2) $$DSC=\frac{2\left|X\cap Y\right|}{\left|X\right|+\left|Y\right|}$$

(3) $$JSC=\frac{\left|X\cap Y\right|}{\left|X\cup Y\right|}$$

where *X* represents the standard segmentation of the temporal lobe delineated by the radiation oncologist and *Y* represents the predicted segmentation by the U-Net model. |X∩Y| denotes the overlapping parts. The value of DSC ranges from 0 to 1, and the closer the value is to 1, the better the model prediction is and *vice versa*.

As shown in Equations (4) and (5), PV and SE are calculated.


(4) $$PPV=\frac{TP}{TP+FP}$$

(5) $$SE=\frac{TP}{TP+FN}$$

where TP denotes temporal lobe pixel points that are correctly predicted, FP denotes background pixel points that are incorrectly predicted as the temporal lobe, and FN denotes temporal lobe pixel points that are predicted as background.

HD is calculated as shown in Equation (6).


(6) H(X,Y)=max(h(X,Y),h(Y,X))

where $h(X,Y)=\underset{x\in X}{\max}\underset{y\in Y}{\min}\left|x-y\right|$ , $h(Y,X)=\underset{y\in Y}{\max}\underset{x\in X}{\min}\left|y-x\right|$ ; the smaller the value of HD, the better the model prediction result.

### 2.6 Model environment and parameters

In the study, TensorFlow software version 2.4.0 (Google Brain Team, 2015; Mountain View, CA, USA) and Keras software version 2.4.3 (Chollet, 2015) were used to build the model and Python 3 (Van Rossum and Drake, 2009) was employed to program it. The operating system used in the study is the Windows 10 64-bit operating system (Microsoft Corp., Redmond, WA, USA) with the following hardware: central processing unit (CPU), Intel Core i9-10900 KF @ 3.70 GHz (Intel Corp., Santa Clara, CA, USA); graphics card, NVIDIA GTX3090 24 G (NVIDIA Corp., Santa Clara, CA, USA); and 128 GB memory. Model hyperparameters, as shown in **Table 1**, were selected from the best results according to the experimental conditions. (Batch Size: the number of input images per iteration. Epoch: the batch to be trained. Image Size: the input size of the image. Learning Rate: the initial learning rate using exponential decay. Decay Steps: the number of steps that have been experienced for a learning rate decay. Decay Rate: the learning rate decay coefficient).

**Table 1:** Network training parameters.

Loss	Batch Size	Epoch	Image Size	Learning Rate	Decay Steps	Decay_Rate
DL	2	120	512 × 512	3e-4	400	0.96
GDL	2	120	512 × 512	1e-3	400	0.96
TL	2	120	512 × 512	1e-3	400	0.96
FTL	2	120	512 × 512	2e-3	500	0.96
Our Loss	2	120	512 × 512	5e-4	600	0.96

### 2.7 Statistical and plotting methods

In this study, statistical analysis was performed using the EXCEL function, and the measures were expressed as mean ± standard deviation.

## 3 Results

In this study, the U-Net model framework based on five different loss functions was used to predict the test set and measured by the related evaluation metrics. The results, shown in **Table 2**, reveal that with the U-Net model based on FGD-BCEL, the DSC is 0.88, which is improved compared with the DSCs of the U-Net models based on DL, GDL, TL, and FTL loss functions, respectively. The improvement between FGD-BCEL and TL is the largest with an improvement value of 0.07. With the U-Net model based on FGD-BCEL, the standard deviation for the DSCS is smaller than those for all the four other loss functions, indicating that the U-Net model based on FGD-BCEL presents less difference among different CT slices. The results of JSC are consistent with those of DSC, with the difference between FGD-BCEL and TL being 0.09. For the PPV, the U-Net model based on FGD-BCEL has no significant improvement compared with the U-Net model based on Dice loss function, but has different degrees of improvement compared with GDL, TL, and FTL loss functions. For the SE, except for the U-Net model based on Dice loss function, the U-Net model based on FGD-BCEL has decreased value compared with the U-Net model based on other loss functions. The HD of the FGD-BCEL-based U-Net model is 4.10, which is higher than that of the U-Net model based on the other loss functions. In summary, it can be concluded that the FGD-BCEL-based U-Net model can better segment the temporal lobe on the localization CT.

**Table 2:** Assessment indices of test set.

	DSC	JSC	PPV	SE	HD
DL	0.87 ± 0.11	0.78 ± 0.11	0.90 ± 0.10	0.87 ± 0.13	4.11 ± 0.75
GDL	0.87 ± 0.10	0.78 ± 0.11	0.88 ± 0.12	0.89 ± 0.12	4.13 ± 0.78
TL	0.81 ± 0.15	0.70 ± 0.17	0.75 ± 0.17	**0.92 ± 0.14**	4.77 ± 0.98
FTL	0.87 ± 0.09	0.78 ± 0.11	0.86 ± 0.12	0.91 ± 0.10	4.20 ± 0.81
Our Loss	**0.88 ± 0.05**	**0.79 ± 0.08**	**0.90 ± 0.10**	0.88 ± 0.10	**4.10 ± 0.82**

* Bold, optimal value.

**Figure 2:**
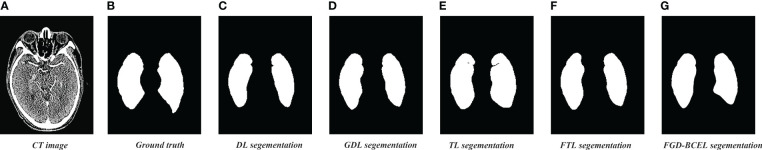
Results of temporal lobe segmentation by the U-Net model based on five loss functions. **(A)** Standard radiotherapy localization CT image (CT image). **(B)** Mask map (Ground truth) sketched by the experts. **(C)** Temporal lobe segmented by the U-Net model based on DL loss function. **(D)** Temporal lobe segmented by the U-Net model based on GDL loss function. **(E)** Temporal lobe segmented by the U-Net model based on TL loss function. **(F)** Temporal lobe segmented by the U-Net model based on FTL loss function. **(G)** Temporal lobe segmented by the U-Net model based on FGD-BCEL loss function.

**Figure 3:**
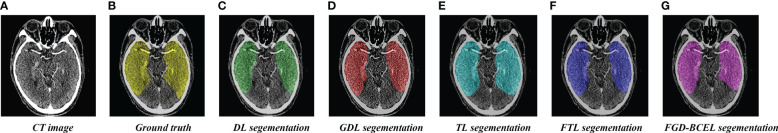
Coverage maps of temporal lobe segmented by the U-Net model based on five loss functions. **(A)** Standard radiotherapy localization CT map. **(B)** Coverage map of the standard temporal lobe (Ground truth) sketched by the experts. **(C)** Coverage map of the temporal lobe segmented by the U-Net model based on the DL loss function. **(D)** Coverage map of the temporal lobe segmented by the U-Net model based on the GDL loss function. **(E)** Coverage map of the temporal lobe segmented by the U-Net model based on the TL loss function. **(F)** Coverage map of the temporal lobe segmented by the U-Net model based on the FTL loss function. **(G)** Coverage map of the temporal lobe segmented by the U-Net model based on the FGD-BCEL loss function.

**Figure 4:**
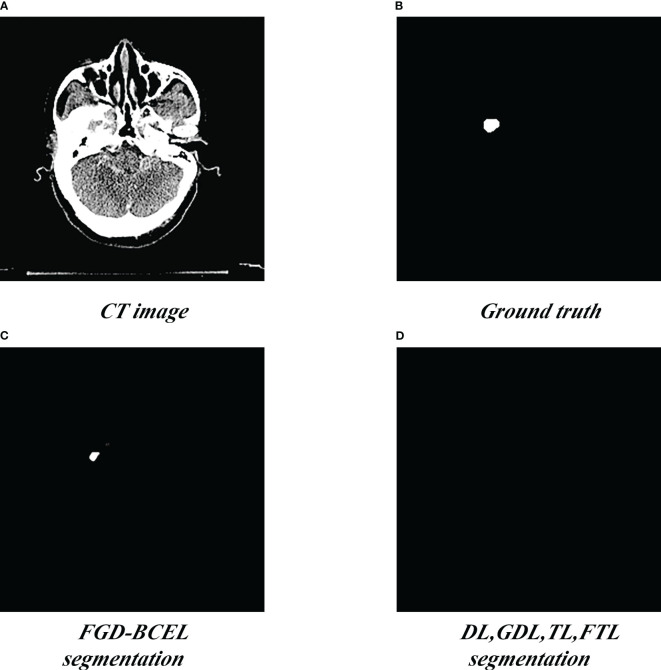
Segmentation of the superior pole of the temporal lobe segmented by the U-Net model based on five loss functions. **(A)** Standard radiotherapy localization CT image (CT image). **(B)** Mask map (Ground truth) by the experts. **(C)** Segmented superior temporal lobe pole maps from the U-Net model based on the FGD-BCEL loss function. **(D)** Segmented superior temporal lobe pole maps from the remaining four loss functions (with the four loss functions, the U-Net failed to segment the superior temporal lobe).

The prediction results of the U-Net model based on different loss functions are shown in **Figures 2**–**4**. The results show that the segmented temporal lobes by the U-Net model based on FGD-BCEL is morphologically more similar to those by the experts. The over- and under-segmentation of the temporal lobe is lessened. Meanwhile, the figure shows that the U-Net model based on DL, GDL, TL, and FTL loss functions cannot, to some extent, perform accurate identification for the tiny structures in the superior and inferior poles of the temporal lobe, probably due to the number of data samples. In contrast, the FGD-BCEL-based U-Net model can effectively perform the prediction of the superior and inferior temporal lobes with a limited number of samples. Therefore, the qualitative results show that the FGD-BCEL-based U-Net model presents less over- and under-segmentation of the temporal lobe besides the effective segmentation of the temporal lobe.

**Figure 5:**
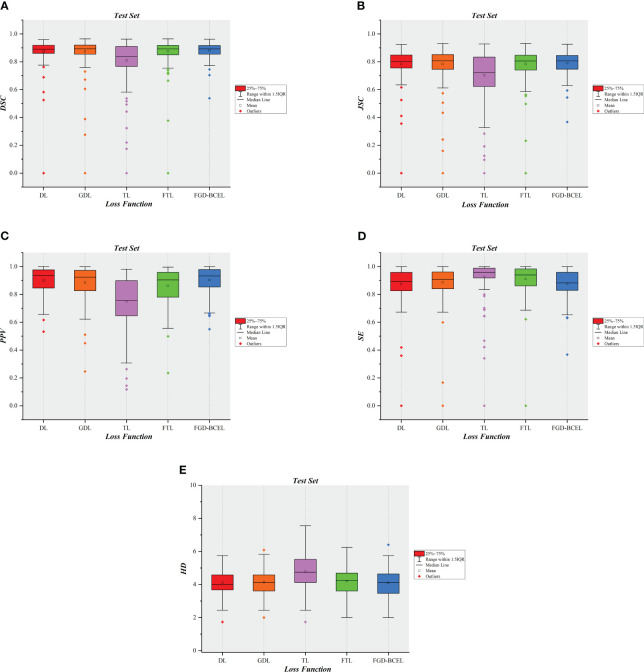
Box plot diagrams in the test set. **(A)** Box plot diagram of DSC in the test set. **(B)** Box plot diagram of JSC in the test set. **(C)** Box plot diagram of PPV in the test set. **(D)** Box plot diagram of SE in the test set. **(E)** Box plot diagram of HD in the test set.

To show the results of the test set more visually, we plotted the results of evaluation metrics about the five loss functions, as shown in **Figure 5** and **Supplementary Figures 2–6**. The box plots indicate that the U-Net model based on FGD-BCEL loss function has fewer outliers or outliers are closer to the median in most evaluation metrics. This result also demonstrates that the U-Net model based on FGD-BCEL has less variability in the accuracy of segmentation results for different CT slices.

## 4 Discussion

In this study, we introduced a novel loss function called FGD-BCEL and applied it to train the U-Net model for medical image segmentation. Additionally, we conducted a comparative analysis of four commonly used medical loss functions to evaluate the performance of FGD-BCEL specifically in segmenting the temporal lobe. The FGD-BCEL loss function addressed two key issues. Firstly, it assigns different weights to each sample, which is employed by FL and GDL. This approach allowed the model to emphasize the foreground target values during training, effectively mitigating the issue resulting from class imbalance, commonly encountered in medical image segmentation. By giving more weights to the foreground regions, the model learned to segment the target more accurately. Secondly, the FGD-BCEL loss function incorporated BCE loss to address the issue of training instability. The introduction of the BCE loss function reduced oscillation during the training process, ensuring stability and consistency of the model, leading to improved segmentation accuracy.

The quantitative results show that except for SE, the other quantitative evaluation indexes of the U-Net model based on the FGD-BCEL loss function are better than those of DL, GDL, TL, and FTL. Simultaneously, the box plot results for each evaluation metric reveal that the U-Net model, which incorporates the FGD-BCEL function, displays a decreased number of outliers or outliers that are closer to the median across the majority of evaluation metrics. Moreover, previous studies have demonstrated that segmentation results meeting the basic criteria are achieved when the DSC exceeds 0.70 (22, 23). All the DSCs for the U-Net models based on all the loss functions used in the study exceed this threshold, among which the DSC value of the U-Net model based on the FGD-BCEL loss function is 0.88, and the U-Net model based on the FGD-BCEL loss function is capable of effectively segmenting the temporal lobe on localized CT images for radiotherapy. The comprehensive analysis of quantitative and box plot results consistently demonstrates that FGD-BCEL exhibits superior performance in segmenting the temporal lobe on localized CT images for radiotherapy with minimal segmentation variations among different slicers, indicating excellent generalization and robustness of the model. This improved performance may be attributable to the incorporation of a weight adjustment mechanism in FGD-BCEL, on the basis of GDL. By regulating the weights assigned to each pixel, the model effectively adapts to the characteristics of the segmentation task, leading to improved segmentation accuracy and performance.

Meanwhile, the qualitative analysis reveals that the U-Net model based on the FGD-BCEL loss function produces, to a certain extent, less over- and under-segmentation than the U-Net model based on DL, GDL, TL, and FTL in automatic delineation of the temporal lobe. Remarkably, it is observed that the four loss functions used as comparison are unable to effectively segment the smaller structures situated in the superior and inferior poles of the temporal lobe. In contrast, the FGD-BCEL loss function demonstrates successful segmentation of these structures. The inability of the other four loss functions to handle these regions can be attributed to their gradient instability, resulting in instable and inaccurate segmentation. The proposed loss function in this study, which incorporates the cross-entropy loss, contributes to a more stable training process, thus enabling effective segmentation. This finding further reinforces the advantage of FGD-BCEL in accurately segmenting small structures.

Furthermore, we compared the segmentation results of this study with other studies on temporal lobe segmentation of nasopharyngeal or head and neck tumors, as shown in **Table 3**. Liu et al. (24) proposed a loss function called TELD-loss for automatic segmentation of the OARs for nasopharyngeal and lung cancer, and the results showed that the mean DSC values of the temporal lobes on the left and right side were 0.7873 and 0.5969, respectively. Compared with them, the resultant value in this study was 0.88, which has a sufficient improvement. Yang et al. used three different deep learning models based on focal loss to automatically segment OARs for NPC. The results showed that the DSCs of the left and right temporal lobes produced by the U-Net model based on focal loss were only 0.58 and 0.61, respectively (25). Similar studies include the work by Mu et al. (26). They proposed an improved loss function by combining the cross-entropy loss and the Dice coefficient, which was applied to segment OARs in the head and neck region. The results showed that their method achieved DSCs of 0.831 and 0.853 for the left and right temporal lobes, respectively. However, compared to the FGD-BCEL loss function used in this study, their segmentation results exhibited inferior performance in both temporal lobes. Furthermore, Peng et al. (27) introduced a novel loss function called body-inside loss to replace the commonly used SoftMax cross-entropy loss (SCE) in training the U-Net model. Their approach achieved similar results to this study in temporal lobe segmentation, with an average DSC of 0.88. To address the issue of class imbalance in segmenting OARs in the head and neck region, Wang et al. (28) proposed a loss function named Boundary Loss based on the boundary loss mechanism. Their method achieved DSCs of 0.848 and 0.841, as well as HDs of 11.32 and 13.58 for the left and right temporal lobes, respectively. However, compared to this study, our proposed FGD-BCEL loss function achieved better performance with a DSC of 0.88 and an HD of 4.10. Additionally, Sun et al. (29) combined batch dice loss, spatially balanced focal loss, and cross-entropy loss to address the issue of class imbalance. Their results showed DSCs of 0.8730 and 0.8699 for the left and right temporal lobes, respectively, which were lower than the results obtained by the FGD-BCEL loss function proposed in this study. In conclusion, the FGD-BCEL loss function proposed in this study exhibits significant superiority in segmenting the temporal lobe on localized CT images for radiotherapy.

**Table 3:** References related to the results of temporal lobe segmentation with different loss functions.

References	Basic model	Patients	Loss function	DSC_mean_ (%)	HD_mean_ (mm)
Liu et al. (24)	CLAF-CNN	50	TELD-loss	69.21	None
Yang et al. (25)	U-Net	147	Focal loss	59.50	4.75
Mu et al. (26)	SE_Residual Block+V-Net	50	Cross entropy + Dice loss	84.20	None
Peng et al. (27)	U-Net	310	Body-inside loss	88.00	None
Wang et al. (28)	Ua-Net	170	Boundary loss	84.47	12.45
Sun et al. (29)	U-Net	112	Batch dice loss + spatially balanced focal loss + cross entropy loss	87.15	None

The values of DSC and HD were the average of the corresponding evaluation index values of the left and right temporal lobes.

In summary, the U-Net model based on our proposed loss function can effectively improve the segmentation results of the temporal lobe, but the box plots show that the U-Net model based on the FGD-BCEL loss function still has some outliers, which may be due to the lack of sample size and small volume of the upper and lower poles of the temporal lobe, thus making it difficult to segment. In further studies, the dataset needs to be increased further. Meanwhile, the segmentation results of the U-Net model based on the FGD-BCEL loss function still have some over- and under-segmentation, and future attempts should be made to incorporate the residual or attention module to further improve the segmentation effect.

## 5 Conclusion

In summary, the U-Net model based on the FGD-BCEL loss function can automatically outline the temporal lobe on localized CT images for radiotherapy, and it can effectively alleviate the over- and under-segmentation of the U-Net model compared with the other four loss functions. However, some over- and under-segmentation still exists, and further improvement is needed in the future.

## Data availability statement

The datasets generated and/or analyzed during the current study are not publicly available due protection of patient privacy but are available from the corresponding author on reasonable request. Requests to access the datasets should be directed to YY, yiyangrt@126.com.

## Ethics statement

This study was approved by the Ethics Committee of Yunnan Cancer Hospital (KYLX2022190). All the methods in this article are in accordance with the relevant guidelines and regulations. Written informed consent for participation was not required for this study in accordance with the national legislation and the institutional requirements.

## Author contributions

XW and BL conceived and designed the study and wrote the manuscript. BZ, MY, XH, and WH delineated or guided the delineation of ROI area. TL compiled the data. XW wrote the code and provided technical support. YY and DX provided data or reviewed articles. All authors contributed to the article and approved the submitted version.
