# Supplementary material for: Application of FGD-BCEL loss function in segmenting temporal lobes on localized CT images for radiotherapy
Source: Front Oncol. 2023 Oct 5;13:1204044. doi: 10.3389/fonc.2023.1204044 (PMC10585164; doi:10.3389/fonc.2023.1204044)
Supplement: Supplementary file 1 [file Image_1.pdf]

**Supplementary Figure 1** Standard data set of temporal lobes. (a). CT image (b). Ground truth (c). temporal lobes drawn in 3D

**Supplementary Figure 2** Box plot diagram of DSC in the test set
**Supplementary Figure 3** Box plot diagram of JSC in the test set
**Supplementary Figure 4** Box plot diagram of PPV in the test set.
**Supplementary Figure 5** Box plot diagram of SE in the test set.

**Supplementary Figure 6** Box plot diagram of HD in the test set.

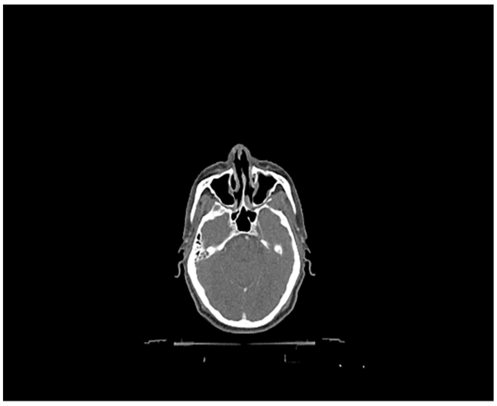

***a.CT Image***

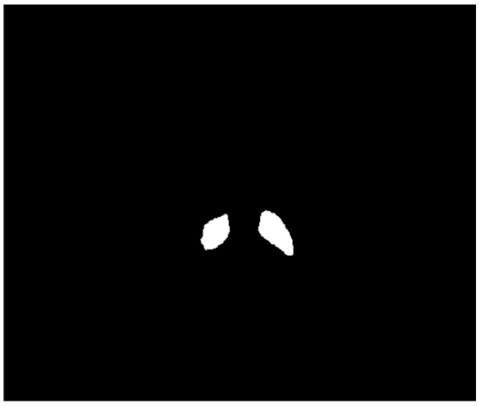

***b.Ground Truth***

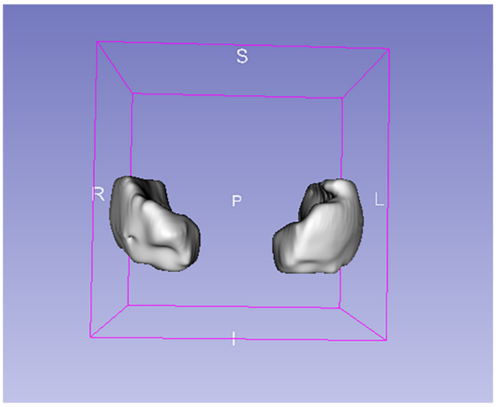


***c. 3D Image***

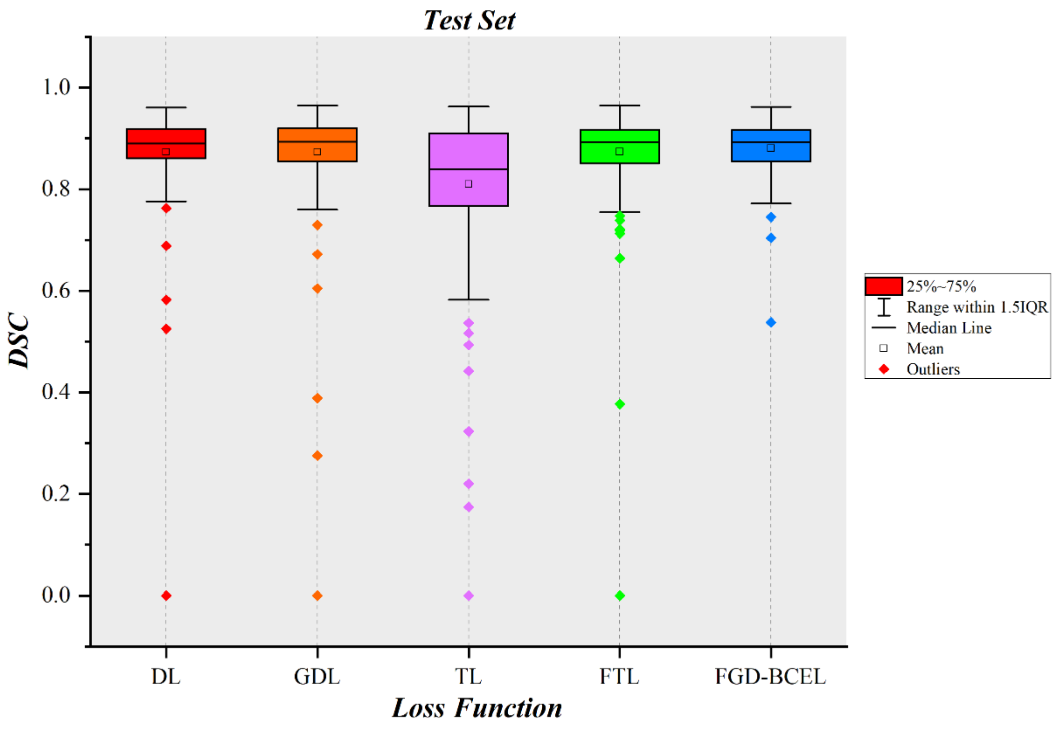
Test Set
DSC
1.0
0.8
0.6
0.4
0.2
0.0
DL
GDL
TL
FTL
FGD-BCEL
Loss Function
25%~75%
Range within 1.5IQR
Median Line
Mean
Outliers

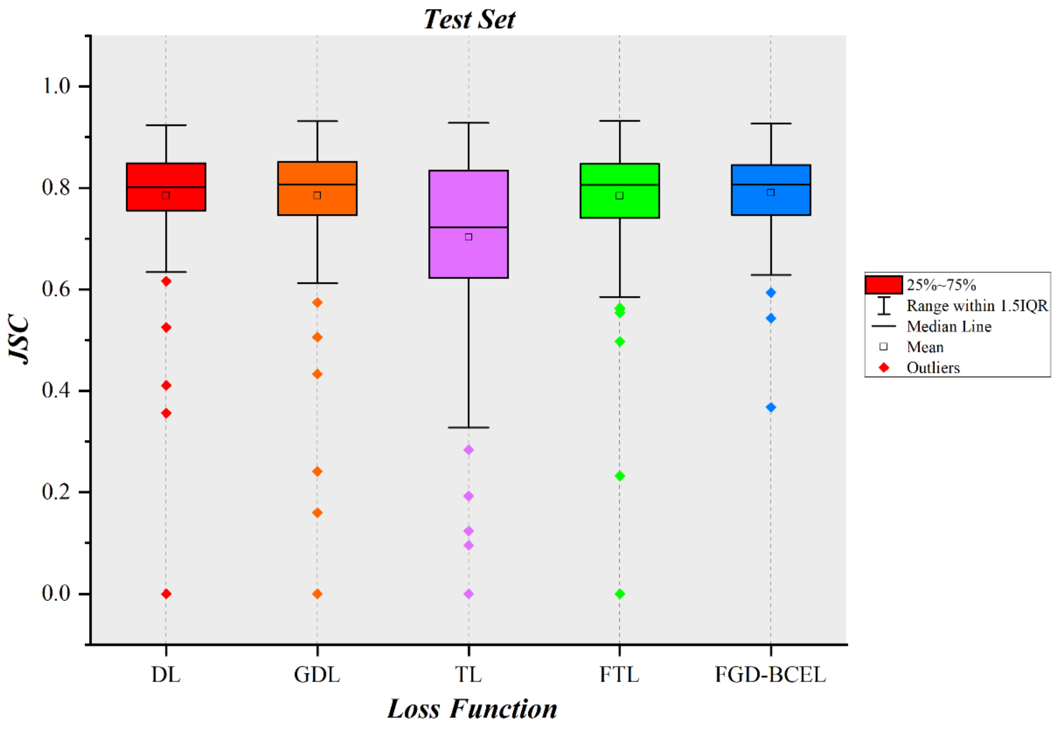
Test Set
JSC
1.0
0.8
0.6
0.4
0.2
0.0
DL
GDL
TL
FTL
FGD-BCEL
Loss Function
25%~75%
Range within 1.5IQR
Median Line
Mean
Outliers

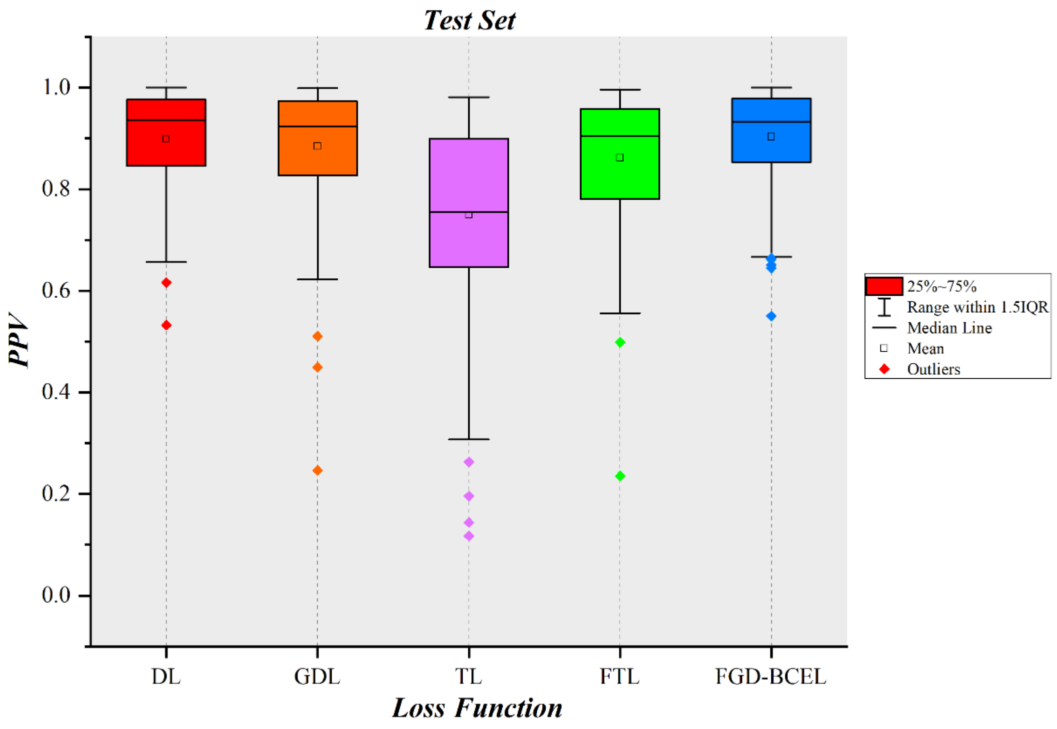

Test Set
PPV
1.0
0.8
0.6
0.4
0.2
0.0
DL
GDL
TL
FTL
FGD-BCEL
Loss Function
25%~75%
Range within 1.5IQR
Median Line
Mean
Outliers


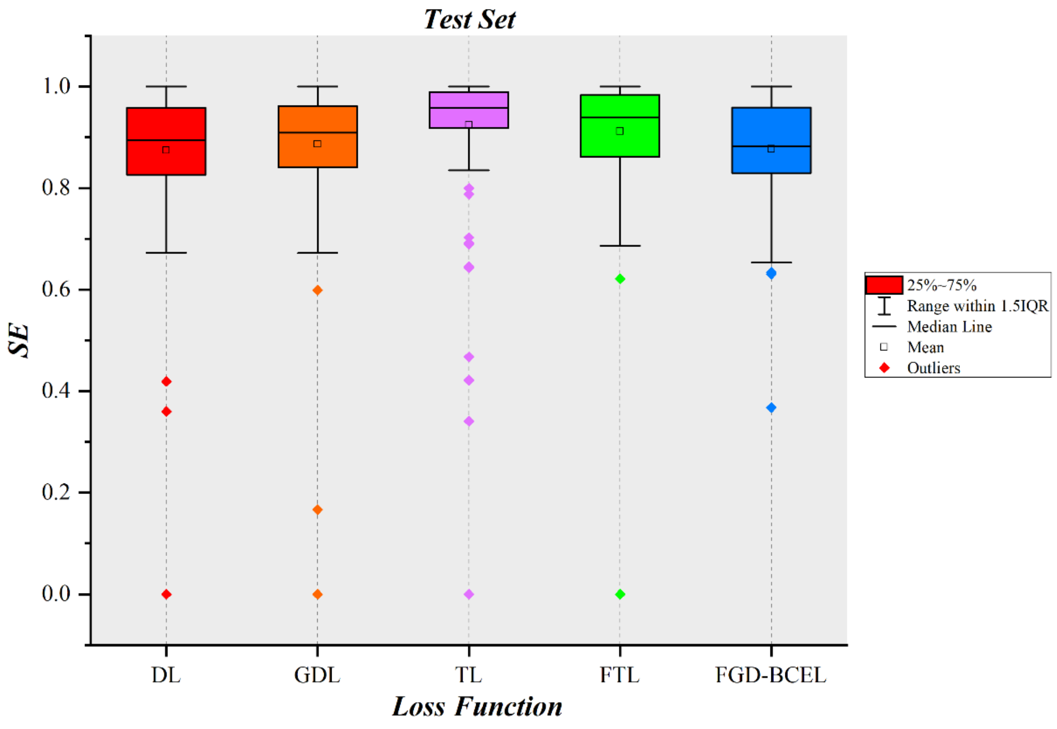

Test Set
SE
1.0
0.8
0.6
0.4
0.2
0.0
DL
GDL
TL
FTL
FGD-BCEL
Loss Function
25%~75%
Range within 1.5IQR
Median Line
Mean
Outliers

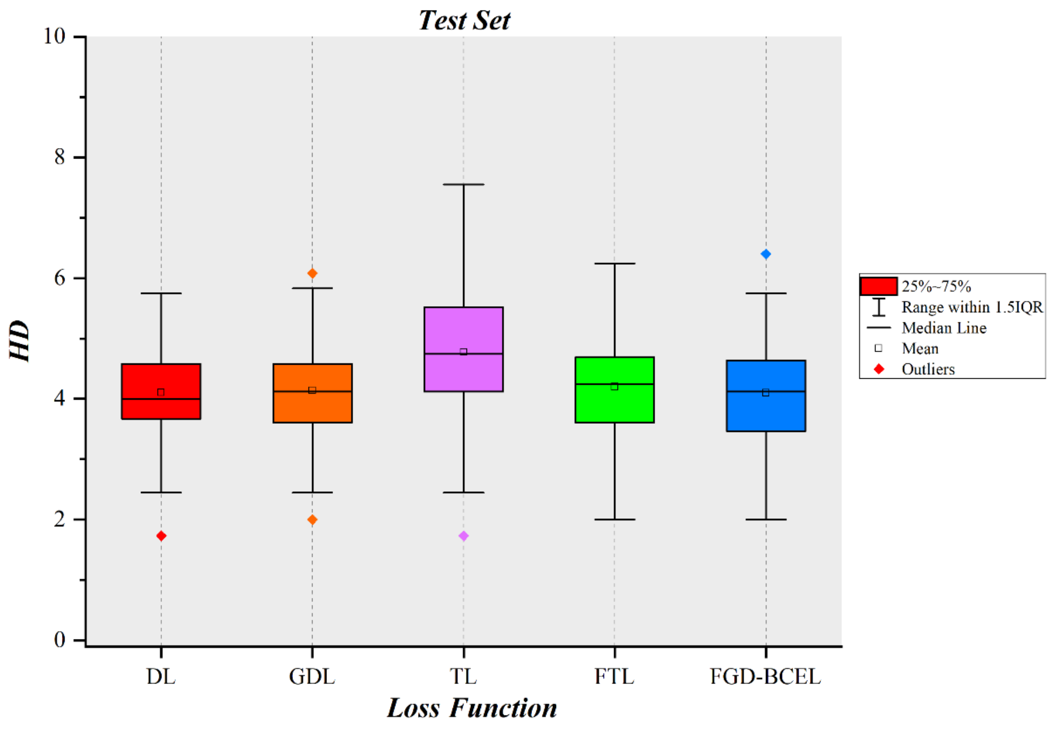

Test Set
10
8
6
4
2
0
HD
DL
GDL
TL
FTL
FGD-BCEL
Loss Function
25%~75%
Range within 1.5IQR
Median Line
Mean
Outliers
